# Liquid Chromatography-Tandem Mass Spectrometry for Analysis of Intestinal Permeability of Loperamide in Physiological Buffer

**DOI:** 10.1371/journal.pone.0048502

**Published:** 2012-11-08

**Authors:** Miriam S. Rubelt, Salah Amasheh, Thomas Grobosch, Christoph Stein

**Affiliations:** 1 Department of Anesthesiology, Charité, Campus Benjamin Franklin, Berlin, Germany; 2 Institute of Clinical Physiology, Charité, Campus Benjamin Franklin, Berlin, Germany; 3 Laboratoriumsmedizin and Toxikologie, Labor Berlin - Charité Vivantes GmbH, Berlin, Germany; Biological Research Centre of the Hungarian Academy of Sciences, Hungary

## Abstract

Analysis of *in vitro* samples with high salt concentrations represents a major challenge for fast and specific quantification with liquid chromatography-tandem mass spectrometry (LC-MS/MS). To investigate the intestinal permeability of opioids *in vitro* employing the Ussing chamber technique, we developed and validated a fast, sensitive and selective method based on LC–MS/MS for the determination of loperamide in HEPES-buffered Ringer's solution. Chromatographic separation was achieved with an Atlantis dC18 column, 2.1 mm×20 mm, 3 µm particle size and a gradient consisting of methanol/0.1% formic acid and ammonium acetate. The flow rate was 0.7 ml/min, and the total run time was 3 min. For quantification, two mass transitions for loperamide and a deuterated internal standard (methadone-d_3_) were used. The lower limit of loperamide quantification was 0.2 ng/ml. This new LC-MS/MS method can be used for the detection of loperamide in any experimental setup using HEPES-buffered Ringer's solution as a matrix compound.

## Introduction

**Figure 1:**
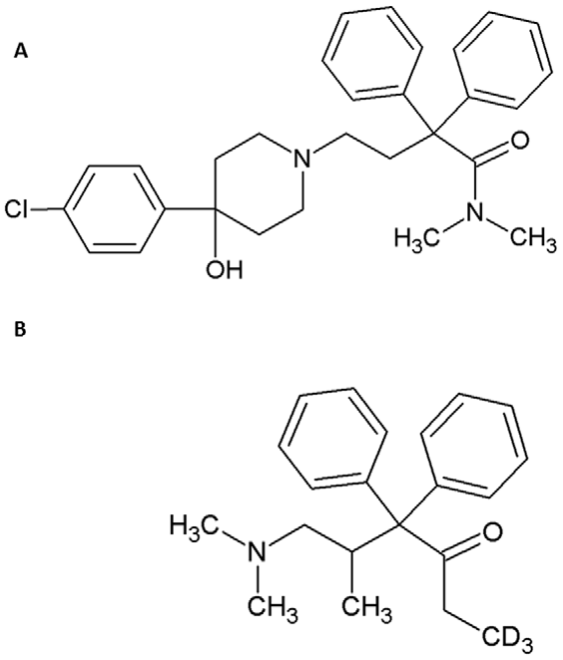
Chemical structures of the target analyte loperamide (A) and the internal standard methadone-d_3_ (B).

Traditionally, opioids have been viewed as prototypes of centrally acting analgesics. However, opioid receptors were also detected on peripheral sensory nerve terminals and were shown to mediate potent analgesic effects, particularly in inflamed tissues [1]. In fact, animal studies have demonstrated that a large proportion (50–100%) of the antinociceptive effects produced by systemically administered opioids can be mediated by peripheral opioid receptors [2]–[7] and human studies indicate that opioid agonists that do not readily enter the central nervous system (CNS) can have the same analgesic efficacy as conventional opioids [8]. In search of opioid ligands that selectively activate peripheral opioid receptors without entering the CNS, we began to study loperamide (Fig. 1A), a synthetic piperidine derivative which has long been used to control diarrhea [9], [10]. Loperamide has low oral bioavailability because of its low absorbance rate from the gut. Similarly, it does not readily pass the blood brain barrier because it is a substrate of the efflux membrane transporter P-glycoprotein (P-gp) [11], [12]. More recently, it has been shown that systemically (subcutaneously) administered loperamide can inhibit inflammatory pain *via* activation of peripheral opioid receptors in rodents [5]. However, in the clinical setting it would be highly desirable to administer loperamide by the oral route. To eventually reach opioid receptors in peripheral inflamed tissues, orally administered loperamide must first permeate the intestinal epithelium and enter the blood stream.

In line with the “3R” (“Refine, Reduce, Replace”) concept to decrease the number of animal experiments [13]–[15], we aim to initially assess the intestinal transport of loperamide *in vitro*. To this end, we employ the human colon cell line Caco-2 in combination with the Ussing chamber technique. Caco-2 cells form a confluent monolayer consisting of columnar and polarized epithelial cells with a microvillous apical membrane, and tight junctions in the apicolateral membrane linking adjacent cells together and determining paracellular permeability [16]–[18]. The Ussing chamber contains buffered Ringer's solution and the permeation of the cell layer by compounds is assessed by measuring their concentration in the basolateral versus apical chamber. To quantify loperamide, we aim to use liquid chromatography coupled with tandem mass spectrometry (LC-MS/MS). This has higher intraclass correlation coefficients and lower coefficients of variation than radioimmunological methods [19], and provides high specificity as each analyte produces a distinct measurable signal [20]. However, so far LC-MS/MS methods are only available for detection of loperamide in serum and liquor [21]–[26] but not in buffered Ringer's solution. Ion suppression is one of the major problems for the detection of analytes in urine, plasma or buffer solutions due to co-elution of analytes or matrix components [27]. This can significantly reduce or enhance the ionization of the analyte in consequence of the low and/or fluctuating signal intensity in the MS system and therefore falsify the quantification. Especially solutions like the organic 4-(2-hydroxyethyl)-1-piperazineethanesulfonic acid (HEPES)-buffered Ringer's solution are predestined to induce such ion suppressions because of their high amount of salts and glucose. Since it is challenging to overcome these problems some researchers named this as a specific type of an art [28]. As there is no universal solution to end up with these problems, every protocol needs its own settings to avoid matrix effects. Reducing the risk of ion suppression effects caused by solvents like HEPES- buffered Ringer's solution is possible, but it needs careful optimization of sample preparation, chromatography and calibration techniques [29]. Therefore, we developed a new specific sample preparation protocol to exclude a possible ion suppression/enhancement and to avoid contamination of the MS system. We used two mass transitions and a deuterated internal standard (IS) (methadone-d_3_) (Fig. 1B). We selected methadone-d_3_ as internal standard, because it bears respectable structural similarity to loperamide and and therefore allowed a fast and streamlined method development. Furthermore we checked already published literature on this topic and found additional publications which also used methadone-d_3_ as well for an internal standard [21], [23]. These findings convinced us that methadone-d_3_ is an appropriate internal standard for the detection of loperamide.

## Materials and Methods

### Reagents and chemicals

All chemicals were, unless otherwise stated, obtained from Sigma-Aldrich (Munich, Germany). Deuterated methadone (methadone-d_3_), purchased from Cerilliant Corporation (Texas, USA), was found to be a very suitable IS for this application since no interfering signals and no matrix effects occurred during the analyses. For the analytical matrix a HEPES-buffered Ringer's solution (140 mM NaCl, 5.4 mM KCl, 1 mM MgSO_4_, 1.2 mM CaCl_2_, 10 mM HEPES, 10 mM Glucose, adjusted to pH 7.4) was used. Methanol and concentrated formic acid (>98%) were purchased from Fluka (Buchs, Switzerland), ammonium acetate and acetic acid from Merck (Darmstadt, Germany) and acetonitrile from LGC Standards (Wesel, Germany). All chemicals, reagents and solvents were of LC-MS/MS or analytical grade. Deionized water was obtained from an in-house water purification system Aqua RO 5–20 (membraPure, Bodenheim, Germany).

### Column liquid chromatography

The analytical column was an Atlantis dC18 Column, 2.1 mm×20 mm, 3 µm particle size (Waters GmbH, Eschborn, Germany). To prevent rapid deterioration of the analytical column, a Phenomenex C18 guard column 4×3.0 mm (Aschaffenburg, Germany) was used. The temperature of the column oven was 40°C. The mobile phase consisted of a mixture of solvent A: MeOH/H_2_O (97/3, v/v)+10 mM ammonium acetate+0.1% acetic acid; and solvent B: MeOH/H_2_O (10/90, v/v)+5 mM ammonium acetate+0.1% formic acid. The following gradient was used for elution: 0–1.5 min: 95-5% B (linear), 1.5–2.5 min: 5% B, 2.5–3.0 min: 5–95% B (linear). The flow rate was 0.7 ml/min, and the total run time was 3 min.

### HPLC and Mass Spectrometry

**Figure 2:**
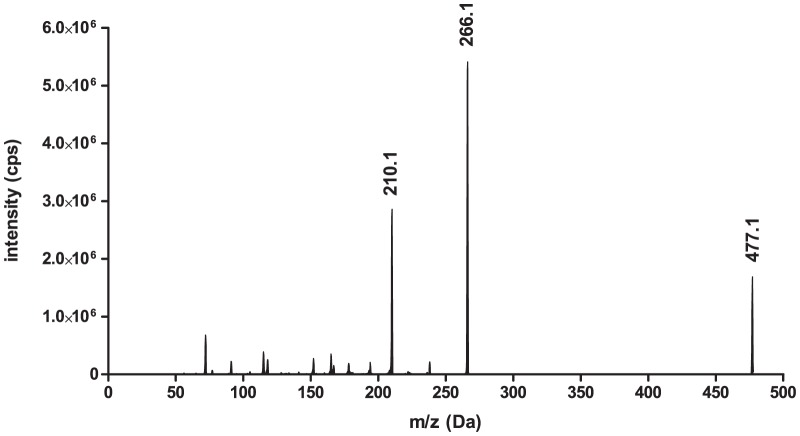
LC-MS/MS spectrum of loperamide, quantifier ion 266.1 m/z and qualifier ion 210.1 m/z.

**Figure 3:**
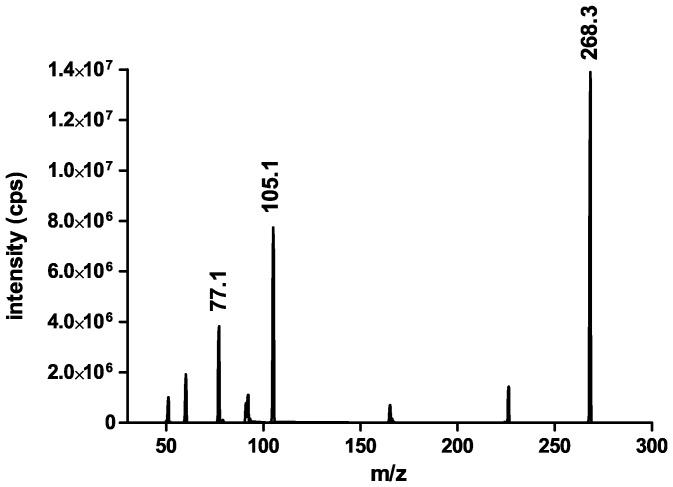
LC-MS/MS spectrum of methadone-d_3_, quantifier ion 268.3 m/z.

**Figure 4:**
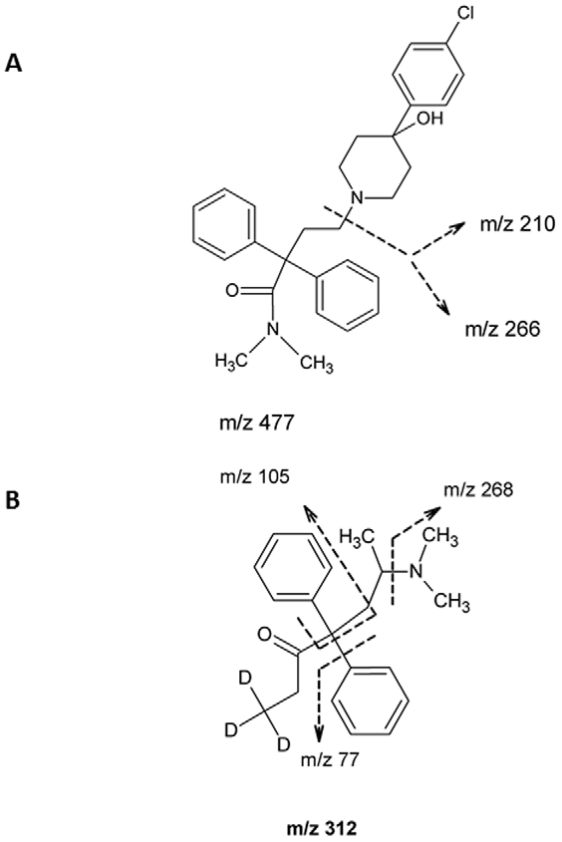
Product ion spectrum of loperamide (A) and methadone-d_3_ (B).

The HPLC system consisted of 2 DGU 20 A degasser, 2 LC-20AD pumps, one optionBox and 2 FCV-14 AH switching valves, a CTO-20A column thermostat and an SIL-HT_A_ auto sampler (all from Shimadzu, Duisburg, Germany). The liquid chromatograph was coupled to a triple quadrupole mass spectrometer (3200 Qtrap; AB SCIEX, Darmstadt, Germany) with a turbo electrospray ion source (EIS) operated in positive ionization mode (ESI+). The source temperature was 450°C and the ion source voltage was set to 4500 V. For loperamide two mass transitions (quantifier and qualifier) were chosen (Fig. 2). For the IS methadone-d_3_ a single mass transition was used (Fig. 3). Compounds were quantified in the multiple reaction mode (ESI+, MRM). The following transitions were monitored (m/z): loperamide: 477.3−>266.3/210.2; IS (methadone-d_3_): 313.3−>268.3. The product ions of loperamide (Fig. 4A) and methadone-d_3_ (Fig. 4B) are shown in figure 4. The retention time for loperamide was 1.81 min and for the IS methadone-d_3_ 1.72 min. The qualifier ion was used to support the distinctive detection of loperamide due to two fragments instead of one. Additionally the qualifier ion was used during the matrix analyzes.

For recording and analyses of the data, Analyst software (version 1.4.2; AB SCIEX, Darmstadt, Germany) was used. To avoid ion suppression effects by the highly concentrated salt solution, a switching valve on board 3200 QTRAP was used, which opened 0.8 min before the initial peak and closed after 2.3 min.

### Sample preparation protocol

A volume of 50 µl IS methadone-d_3_ (50 ng/ml solved in acetonitrile) was added to 100 µl of the sample and briefly vortexed and centrifuged for 5 min at 20,000×g. Subsequently, 100 µl were carefully transferred into a glass vial and measured with an injection volume of 25 µl. Samples with a concentration higher than 100 ng/ml were diluted in HEPES-buffered Ringer's solution to fit the linear range of the standard curve.

### Quantification

To determine the analyte concentration, samples with defined amount of analyte (calibrators) were used to create a loperamide standard curve in HEPES-buffered Ringer's solution. For calculating the loperamide concentration, a linear standard curve based on six different calibrators (1, 5, 10, 25, 50 and 100 ng/ml) and two quality control samples (7.5 and 75 ng/ml) was used. For quantification, the relative peak area of the analyte loperamide was compared to the peak area of the IS. Sample peaks were automatically integrated and concentrations in unknown samples were calculated from the resulting calibration curves (Analyst software, version 1.5.1, AB Sciex, Darmstadt, Germany).

### Validation

The validation of analytical methods is a prerequisite for the quality and comparability of analytical results. The validation of this method was performed following the guidelines published by Peters et al. [30] and the guidelines of the Society of Toxicological and Forensic Chemistry (GTFCh) [31]. It is a widely performed standard validation procedure which is generally employed at the Institute Labor Berlin in the Department of Clinical Toxicology and Pharmacology [32] and other clinical toxicology labs in Germany for the validation of novel methods of specific drug detection.

### Precision and bias

The bias is used as a reference for the difference between actual and desired value and serves as an important tool for the accuracy of quantitative analysis methods.

The bias was calculated with the following equitation:
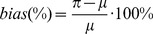

π = mean
μ = reference value

The precision is used to determine the degree to which repeated measurements under unchanged conditions show the same results. It helps to express the reliability of certain measurements [29].

The precision was calculated with the following equation:
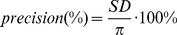

SD = standard deviation

### Specificity

The specificity test is used to make sure that the analyte is clearly identified without interference by other compounds contained in the sample. Therefore individual solutions of the substances (including IS) with a concentration of 1 µg/ml in mobile phase B were prepared (100 µl standard solution [β = 0.01 mg/ml]+900 µl mobile phase B) and analyzed. Two solutions, one with loperamide and one with the IS methadone-d_3_ were measured.

### Selectivity

The analyte should be clearly identifiable without any disturbances of other potentially contaminating substances (metabolites, impurities, matrix, etc.) in the HEPES-buffered Ringer's solution. Therefore, HEPES solutions with no IS were measured twice and the chromatogram analyzed.

### Precision and accuracy

Inter-day precision and accuracy of the method were determined by analyzing 8 quality control (QC) samples from both concentrations on 8 consecutive days. Both sample levels were processed and analyzed once a day. Intra-day precision and accuracy of the method were determined by analyzing 8 QC from both concentrations within one day. Again, the measured concentration values were tested for outliers using the Grubbs test. Outliers were eliminated when criteria fulfilled recommendations for relative standard deviation, and bias values according to [30], [31] were not reached.

### Limits of quantification

The range of operation corresponds to the concentration range of the analyte in the sample which allows a quantitative determination of the analyte with defined accuracy. The lower limit of quantification (LLOQ) was the lowest concentration where relative standard deviation and bias were ≤20%. The calibrator with the lowest analyte concentration (A = 1 ng/ml) was diluted 1∶2, 1∶5 and 1∶10 in HEPES Ringer's solution to determine the LLOQ. The measurement was repeated 8 times and samples were prepared according to the analyzing protocol. The upper limit of quantification (ULOQ) was assumed to be equal to the upper limit of the linear range of the method. To determine the ULOQ a complete series of calibrators was analyzed on 8 consecutive days.

### Matrix effect

A possible effect of coeluting matrix compounds on the ionization of the analyte was investigated by post-column infusion of a standard solution containing loperamide (10 µg/ml in eluent B) and methadone-d_3_ (10 µg/ml in eluent B) via a syringe pump (Harvard Apparatus, Holliston, MA, USA) with a flow rate of 10 µl/min while simultaneously analyzing a blank HEPES-buffered Ringer's solution sample.

### Stability

To investigate freeze/thaw stability, 6 samples from each level (7.5 ng/ml and 75 ng/ml) were subjected to 3 freeze/thaw cycles, each consisting of a 20 h freezing phase and a 20 h thawing phase. Concentrations of these pretreated samples were compared to untreated samples (n = 6). The mean of the pretreated samples had to be within a ±10% interval of the mean of the untreated samples, while the 90% confidence interval was supposed to be within a ±20% interval of the mean of untreated samples.

### Ussing chamber

**Figure 5:**
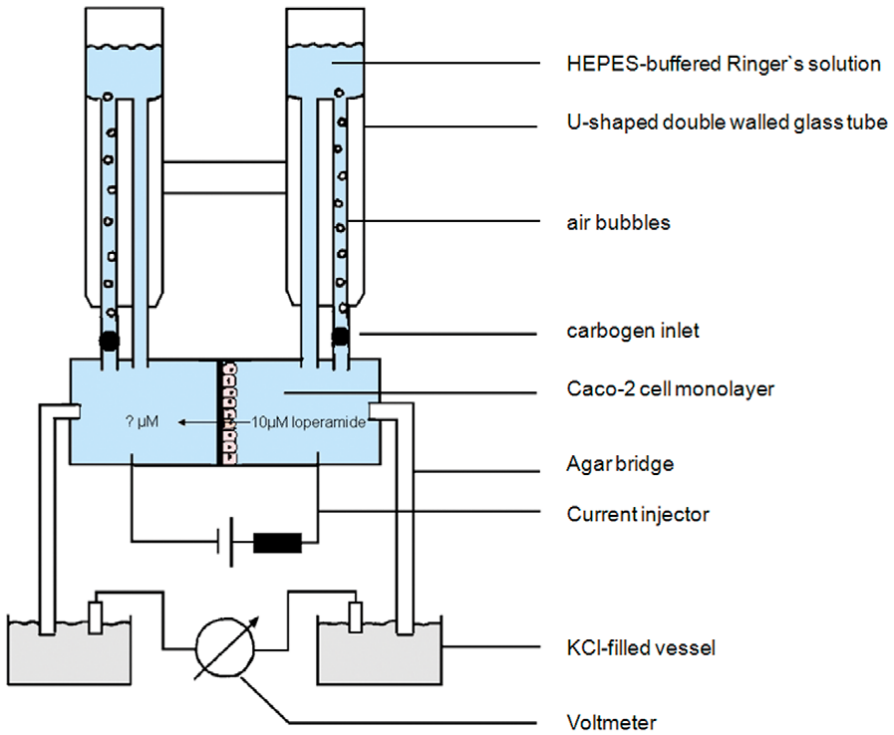
The Ussing chamber setup (modified after Li et al., 2004).

We employed the Ussing chamber technique (Fig. 5) for analysis of epithelial transport and barrier function [33], [34], as described previously [18]. The electrical circuitry allows measurements of resistance (*R*), current (*I*), and voltage (*U*), as well as impedance and capacitance [34]. The chamber consists of two halves separating the basolateral from the apical side of a Caco-2 cell monolayer grown on a permeable support (Fig. 4). Both chamber sides were filled with 5 ml HEPES-buffered Ringer's solution and warmed to 37°C. To ensure circulation and oxygenation, both chamber sides were permanently gassed with a mixture of 95% O_2_ and 5% CO_2_. The system was checked for noise and offset voltages prior to the experiment. Subsequently, the resistance of the empty chambers, a parameter required for correct calculation of resistance and currents, was determined [34]. For controlling and timing of the measurements the software program Analogon (D. Sorgenfrei) was used. Epithelial cells have barrier properties determined by tight junctions [18]. In the intestine, segment-specific barrier properties are determined by specific expression of tight junction proteins [35]. A decrease in electrical resistance can reflect a decrease in tightness of these junctions, and therefore a higher permeability for substances. Using transepithelial voltage (*U*) and current (*I*) the transepithelial resistance (R^t^) was calculated by Ohm's law:
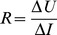



### Cell culture

Caco-2 cells were grown near confluence in monolayer cultures on permeable Millicell HA filters (Millipore; pore size 0.45 µm) at 37°C in an atmosphere of 5% CO_2_ in Minimum Essential Medium (MEM)+GlutaMAXTM (Gibco®, Germany, Karlsruhe) containing 15% (v/v) fetal bovine serum (FBS) and 1% penicillin/streptomycin (PAA Laboratories GmbH, Pasching, Austria). Cell filters with 0.6 cm^2^ surface (∼450000 cells) were used for the Ussing chamber experiments 14 days after cell monolayers reached polarized confluence, giving transepithelial resistances (R^t^) of ∼300 Ω·cm^2^.

### Permeability studies

Loperamide was first solved in ≥99.7% DMSO and then further diluted in HEPES-buffered Ringer's stock solution to the final concentration of 10 µM. Loperamide was first added to the apical side. Afterwards 100 µl samples from both sides were taken in defined time intervals. Beginning at 5 min after the addition of loperamide (time 0) samples were taken from the basolateral side. Each time an equal volume of fresh HEPES-buffered Ringer's solution was replaced. The permeability is defined as the rate of drug transport into the receiver compartment (basolateral side) depending on the drug concentration on the apical side and the area of the cell filter membrane. The following equation was used [36]–[38].
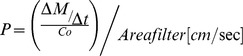

*Δ M/Δ t* rate of drug transport in receiver compartment [ng/sec]
*Δ M* amount of drug on the basolateral side [ng]
*Δ t* time point [sec]
_Co_ concentration of drug added to the apical side [ng/ml]
*AREAfilter* area of the filter membrane [cm^2^]

## Results and Discussion

Sample workup by just adding the IS with a short mixing time and centrifugation time of 5 min proved to be sufficiently fast and easy. We were able to achieve chromatographic separation of all analytes within a 3 min run. Sample preparation with acetonitrile followed by centrifugation of the samples insured a clear solution (due to the precipitation of proteins and salt). This way of sample preparation revealed a standard curve with linear regression coefficients close to 1 (>0.995). All results are related to the mass transition of the quantifier ion.

### Specificity

**Figure 6:**
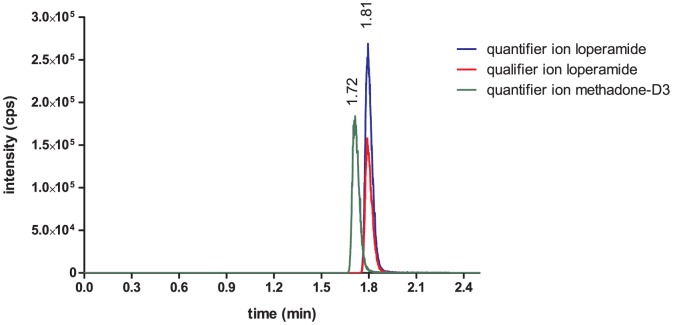
Representative MRM-chromatogram of loperamide (quantifier ion in blue, qualifier ion red) and of the internal standard methadone-d_3_ (green) in HEPES-buffered Ringer's solution.

During the measurements, no interfering signals occurred. This high specificity separates our new method from previous detection methods for loperamide and ensures a appropriate detection of loperamide in HEPES-buffered Ringer's solution. An interfering signal is defined by a signal/noise ratio >10 in a time interval of ±5% of the expected retention time of the particular analyte. In figure 6 a representative MRM-chromatogram of loperamide and the IS methadone-d_3_ in HEPES-buffered Ringer's solution is shown.

### Selectivity

Both chromatograms showed no interfering signals. This indicates that the HEPES-buffered Ringer's solution had no effect on the identification of the analyte loperamide. Therefore HEPES-buffered Ringer's solution which is often used in a great number of cellular experiments, is a suitable matrix compound for the detection of loperamide.

### Precision and accuracy

Inter-day bias for quality control (QC) I was not greater than 13.2% and for QC II not greater than 7.4%. Intra-day bias for QCI did not exceed 7.4% within a precision of 6.6%, and for QCII 14.6% within a precision of 8.2%. Eight values for inter-day and intra-day measurements remained for calculation after the Grubbs test. The conditions of precision and bias <15% were fulfilled for both QCs.

### Measurement range

The LLOQ of loperamide in HEPES Ringer's solution was 0.2 ng/ml. All data points were tested for potential outliers using the F-test (significance level 99%), the goodness of fit was tested according to Mandel [39]. The coefficient of correlation (R) was determined as well. The bias was ±50%, precision was ≤50% and the significance level was 99%. The detection limit of loperamide in HEPES-buffered Ringer's solution was 0.1 ng/ml, within a bias ±20%, precision of ≤20% and a significance level of 99%.

### Matrix effect

**Figure 7:**
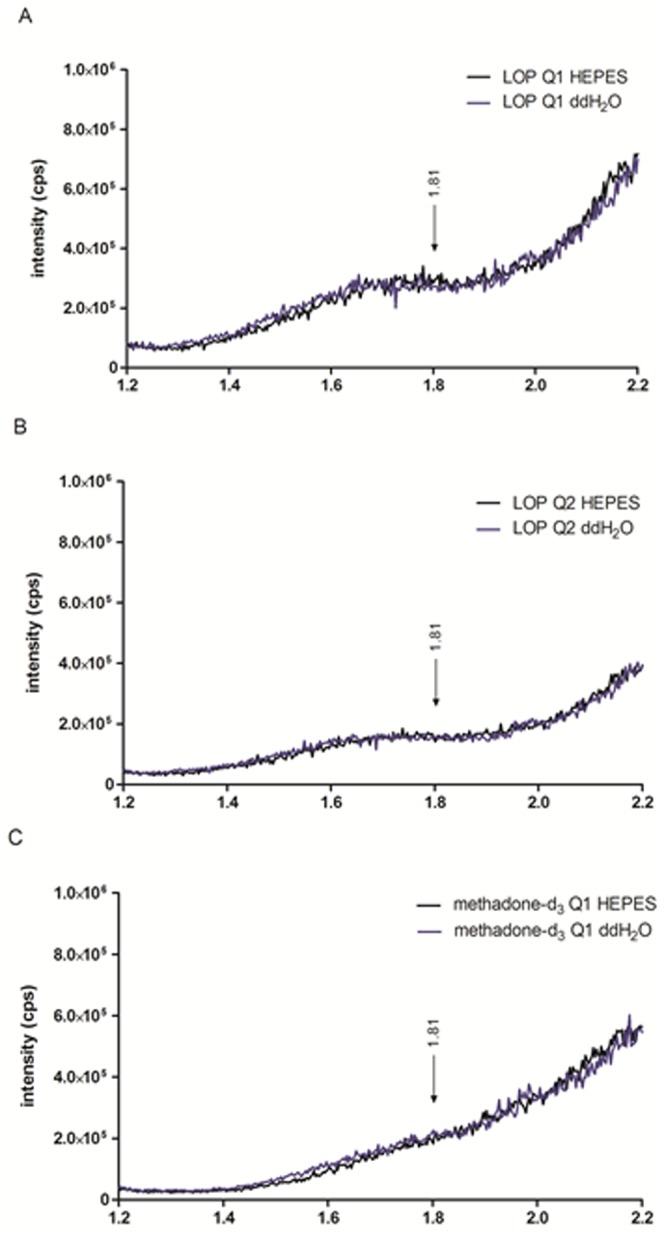
Ion-suppression profiles. Ion chromatograms for qualifier (A) and quantifier (B) of loperamide (LOP) and quantifier of methadone-d_3_ (C), comparison between HEPES-buffered Ringer's solution and blank solution of ddH2O. The arrow indicates where one expects the loperamide peak.

During the post column infusion the signal intensity for loperamide and methadone-d_3_ did not change indicating that no ion suppression or enhancement occurred, the ion-suppression profile is shown in figure 7, qualifier (7A) and quantifier (7B) of loperamide and quantifier of methadone-d_3_ (7C).

### Stability

There was no significant difference between the two untreated QC samples and those subjected to 3 freeze/thaw cycles over the course of at least 6 h. The mean of the pretreated samples was within a ±10% interval of the mean of the untreated samples, while the 90% confidence interval was within a ±20% interval of the mean of untreated samples.

### Permeability studies

**Figure 8:**
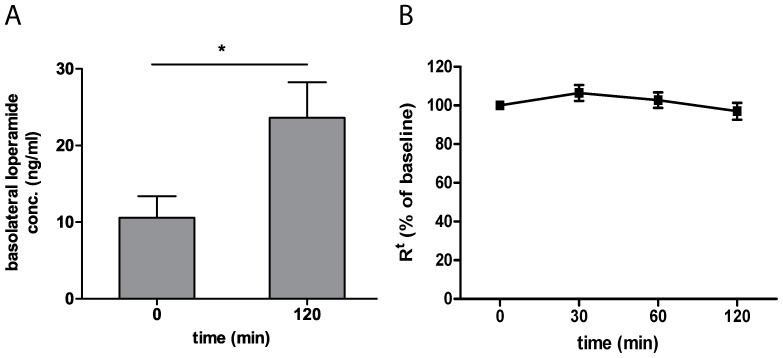
Loperamide concentrations on the basolateral side of the Ussing chamber (n = 4, mean±SEM, student t test p = 0.0164) (A), Transepithelial resistance (R^t^) normalized to baseline values (328±18 Ω·cm^2^) (n = 4, mean±SEM) (B).

After two hours 1/500 of the apical loperamide amount was received on the basolateral side. This concentration was significantly increased in comparison to the values at time 0 (Fig. 8A). The epithelial resistance data showed no significant changes over two hours (Fig. 8B), and the permeability value for loperamide at time point 120 min is 2.72·10^−6^±0.54·10^−6^ cm/sec (n = 4, mean±SEM). Our results are consistent with previous studies on loperamide [40], [41]. However, those studies did not use the Ussing chamber system for transport analyses of charged molecules. Besides, we used a high sensitive, selective and fast LC-MS/MS technique with an easy and rapid sample preparation protocol.

### Conclusion

A fast, selective and sensitive LC-MS/MS method for the quantification of loperamide in HEPES-buffered Ringer's solutions was developed and validated. No interfering signals occurred during the method development and validation which ensures a specific detection of the analyte. The validation data revealed reliable and reproducible results according to the guidelines [30], [31] and covers a loperamide concentration range from 0.2 to 100 ng/ml. The use of two mass transitions as well as specific retention time as criteria for quantification and identification can detect the risk of interferences. Sample preparation is easy to use, cost effective because of the short run time and suitable for high throughput. Aside from the Ussing chamber technique for intestinal epithelial permeability studies, the method described here can be applied for detection of loperamide in any other *in vitro* assay system using HEPES-buffered Ringer's solution.

Furthermore, our approach lays the base for a plethora of novel drug targeting and drug delivery studies, using different cells, tissues and substances. The Ussing chamber technique has the advantage to permit measurements also on charged molecules, as the zero voltage clamp modus abolishes driving forces provided by the cell's endogenous ion transport systems, thus preventing possible artefacts. The HEPES buffer has been established in experiments on a wide variety of epithelial cell models and *in vitro* preparations, providing a stable pH and allowing measurements for extended periods of time [42]. Further advantages of this method are the high specificity and sensitivity even for small amounts of a drug, and the fast and easy sample preparation protocol. Only the final LC-MS/MS detection has to be tuned to the different chemical properties of each analyte. Moreover, *in vivo* approaches can benefit from the established LC-MS/MS detection protocol as well, as further variations of single parameters are marginal compared to the effort of the development of a full detection protocol.
